# Effects of Subthalamic Nucleus Deep Brain Stimulation and Levodopa on Balance in People with Parkinson’s Disease: A Cross Sectional Study

**DOI:** 10.3390/brainsci10100693

**Published:** 2020-09-30

**Authors:** David S. May, Linda R. van Dillen, Gammon M. Earhart, Kerri S. Rawson, Joel S. Perlmutter, Ryan P. Duncan

**Affiliations:** 1 Program in Physical Therapy, Washington University in St. Louis, St. Louis, MO 63108-2212, USA; dsmay@wustl.edu (D.S.M.); vandillenl@wustl.edu (L.R.v.D.); earhartg@wustl.edu (G.M.E.); rawson@wustl.edu (K.S.R.); perlmutterjoel@wustl.edu (J.S.P.); 2 Department of Orthopaedic Surgery, Washington University in St. Louis, St. Louis, MO 63130, USA; 3 Department of Neurology, Washington University in St. Louis, St. Louis, MO 63110, USA; 4 Department of Neuroscience, Washington University in St. Louis, St. Louis, MO 63110, USA; 5 Department of Radiology, Washington University in St. Louis, St. Louis, MO 63130, USA; 6 Program in Occupational Therapy, Washington University in St. Louis, St. Louis, MO 63110, USA

**Keywords:** balance, Parkinson’s disease, deep brain stimulation, levodopa, posture

## Abstract

Subthalamic nucleus deep brain stimulation (STN-DBS) and levodopa are common treatment strategies for Parkinson’s disease (PD). However, the specific effects of these treatment strategies on balance and its components remain unclear. This cross-sectional study of people with PD and STN-DBS compared balance in the treated state (ON-medication/ON-stimulation) and untreated state (OFF-medication/OFF-stimulation) using the Balance Evaluation Systems Test (BESTest). Total BESTest scores from the treated and untreated states were compared to assess overall balance. Scores for the six sections of the BESTest were further compared to assess differences in specific components of balance between treatment conditions. Twenty-nine participants were included (Male: 21, Female: 8, Mean Age ± SD: 65.0 ± 6.9). Total BESTest scores showed improved balance in the treated state compared to the untreated state (Treated: 67.56 ± 10.92; Untreated: 59.23 ± 16.51, *p* < 0.001). Four sections (Stability Limits/Verticality, Anticipatory Postural Reactions, Sensory Orientation, Stability in Gait) of the BESTest significantly improved in the treated state relative to the untreated state, after correcting for multiple comparisons (*p* < 0.05). These results demonstrate that STN-DBS and levodopa improve overall balance and provide a first step toward understanding the effects of these treatment strategies on specific components of balance.

## 1. Introduction

Balance dysfunction, a risk factor for falls, impairs quality of life for people with Parkinson’s disease (PD) [1]. Dopaminergic medication and deep brain stimulation of the subthalamic nucleus (STN-DBS) are common treatment strategies for the cardinal motor signs and symptoms of PD. Levodopa effectively reduces the severity of tremors, bradykinesia, and rigidity, but lacks the same efficacy for reducing balance dysfunction [2,3,4,5], though one study suggests an association with improved balance [6]. The effect of STN-DBS on balance remains unclear. People with STN-DBS show improved overall balance when ON-stimulation, compared to when OFF-stimulation in one small study [7]. The effects of STN-DBS on balance over time are also not known. Some reports suggest short-term or no significant improvement [8,9] in balance following STN-DBS surgery [10], whereas others show a significant decline in automatic postural responses and an increase in falls six months after receiving STN-DBS [11,12]. A reference group of people with PD that did not have STN-DBS did not demonstrate these changes. [11,12].

These ambiguities regarding the effects of STN-DBS on balance may reflect variability of assessment and underscore the need for more comprehensive and consistent balance assessment in this population. Balance is often assessed following STN-DBS through the postural instability and gait difficulty (PIGD) items of the Unified Parkinson’s Disease Rating Scale (UPDRS) or the Movement Disorders Society-Sponsored Revision of the UPDRS (MDS-UPDRS). Others applied a more detailed balance assessment, such as the Berg Balance Scale (BBS), to investigate the effects of DBS [10]. However, all these ratings provide limited insight into the specific components that contribute to balance, such as verticality, reactive postural responses, or stability in gait with or without cognitive influences [13]. Therefore, a more comprehensive clinical assessment will improve our understanding of how levodopa and STN-DBS affect balance [14].

The Balance Evaluation Systems Test (BESTest) provides a comprehensive assessment of balance, covering six balance systems: (I) Biomechanical Constraints, (II) Stability Limits/Verticality, (III) Anticipatory Postural Adjustments, (IV) Postural Responses, (V) Sensory Orientation, and (VI) Stability in Gait. This test is reliable, valid, and accurately predicts prospective fall risk in people with PD [15,16]. Using the BESTest in this population will help determine if and how specific balance systems respond to both levodopa and STN-DBS. The combination of levodopa (ON-medication) and STN-DBS (ON-stimulation) represents a common treated state and is perhaps the most optimal treated state in this population. By comparing balance performance in the most optimal treated state to balance in the least optimal treated state, we can determine whether these strategies effectively treat balance and its components when used in conjunction, or whether balance deficits remain. This knowledge could assist clinicians in tailoring intervention strategies for people with PD following STN-DBS. Therefore, the primary aim of this study was to determine whether total BESTest balance scores differ while ON-medication/ON-stimulation compared to OFF-medication/OFF-stimulation. We hypothesized that total BESTest scores would be higher, indicating better balance performance, in the ON-medication/ON-stimulation condition relative to the OFF-medication/OFF-stimulation condition. 

As an exploratory aim, we also examined if certain sections of the BESTest showed significant changes between the ON-medication/ON-stimulation condition and the OFF-medication/OFF-stimulation condition. Little is known regarding the responsiveness of specific domains of balance to these treatment strategies, but such findings could provide a rationale for therapeutically targeting specific deficits and then testing such strategies in future studies. We speculated that sections (III) Anticipatory Postural Adjustments, (IV) Postural Responses, and (VI) Stability in Gait would show improvement when ON-medication/ON-stimulation compared to OFF-medication/OFF-stimulation as: (1) previous studies report medication improves scores on the BBS, a measure which largely focuses on anticipatory postural adjustments [6]; (2) STN-DBS improves scores on a similar postural response measure [11]; and (3) a combination of STN-DBS and medication improved gait performance during the Dual Task Timed Up and Go [7]. We did not speculate on the sections for (I) Biomechanical Constraints, (II) Stability Limits/Verticality, or (V) Sensory Orientation due to a lack of existing literature.

## 2. Materials and Methods

### 2.1. Participants

This study used cross-sectional participant data from baseline assessments of a pilot randomized clinical trial examining the feasibility and efficacy of physical therapy following STN-DBS surgery [17]. The randomized clinical trial was registered on clinicaltrials.gov (NCT: 03181282). Participants were at least 30 years of age and met the following inclusion criteria: (1) neurologist diagnosed idiopathic PD between Hoehn & Yahr (H&Y) stages II-IV; (2) at least one year post-bilateral STN-DBS; (3) able to attend assessment sessions; and (4) able to provide informed consent. Exclusion criteria included: (1) diagnosis of atypical parkinsonism; (2) evidence of dementia (i.e., Mini-Mental Status Exam (MMSE) score ≤24/30) [18]; or (3) inability to walk ten meters with or without an assistive device. Only participants who were at least one year after DBS surgery were included in this study to ensure that DBS settings were optimized. Written informed consent was obtained from each participant in accordance with the Declaration of Helsinki and the policies and procedures of the Human Research Protection Office at Washington University in St. Louis. This study was approved by the Institutional Review Board at Washington University in St. Louis (201609148). 

### 2.2. Assessments

Balance was assessed by the Balance Evaluation Systems Test (BESTest). The 27-item BESTest is a clinical assessment of balance, with a total possible score of 108 points [15]. Higher scores indicate better balance. The BESTest is highly reliable in people with PD [19] and features six sections corresponding to six components of balance. The total BESTest raw score for each participant was divided by the total possible amount of points (108) to calculate a total percentage score. The raw score for each of the six balance system sections was converted to a percentage score by dividing the raw score by the total possible points for each section.

The BESTest was performed for each participant while in the following conditions: (1) ON-medication/ON-stimulation and (2) OFF medication/OFF-stimulation. These conditions were tested on separate days within one week of each other. The order of testing conditions was randomized. For the ON-medication/ON-stimulation day, ON medication was determined by a participant report of feeling ON during testing and occurred 45–90 min following levodopa intake. The participants’ stimulators remained ON throughout this session. The stimulation parameters were unchanged from those that were optimized clinically by a movement disorders neurologist. For the OFF-medication/OFF-stimulation day, OFF medication was defined as greater than or equal to 12 h since the last intake of anti-PD medication. Participants’ stimulators were turned OFF upon arrival to the laboratory, and testing commenced 45 min later [20]. Stimulators were turned back on upon completion of the testing session. The rater was blinded to the testing condition.

Total levodopa equivalent daily dose (LEDD) was calculated by documenting the amount of each antiparkinsonian drug taken daily by each participant and using a formula to determine the total daily dose of antiparkinsonian medication [21]. The mean and standard deviation of participants’ LEDD values were calculated to characterize the sample. The Movement Disorders Society-sponsored revision of Unified Parkinson’s Disease Rating Scale (MDS-UPDRS) is a valid and reliable clinical assessment for people with Parkinson’s disease [22]. MDS-UPDRS-II and MDS-UPDRS-III were administered to characterize the severity of general parkinsonian motor symptoms in the sample. The MDS-UPDRS-II is a questionnaire pertaining to motor experiences of daily living, with questions regarding activities within the past week [22]. As participants were OFF-medication/OFF-stimulation for a brief time, the MDS-UPDRS-II was only administered once in the ON-medication/ON-stimulation condition. The MDS-UPDRS-III is a general motor examination performed by a clinician or rater [22]. The MDS-UPDRS-III was performed both ON-medication/ON-stimulation and OFF-medication/OFF-stimulation to show motor performance in the optimal treated state and the least optimal treated state.

### 2.3. Statistical Analysis

Means and standard deviations were calculated for participant characteristics, such as age, MDS-UPDRS-III scores, years since PD diagnosis, and months since DBS surgery. A formal power analysis was not conducted specifically for this analysis since these data were taken from baseline measures from a study to assess physical therapy strategies in people with PD and STN-DBS [17]. The alpha level of significance was set at 0.05. Statistical analysis was performed in R software version 3.6.0, R Foundation for Statistical Computing, Vienna, Austria [23]. R code used for data analysis is available at https://github.com/dsmay11/R-Code-STN-DBS-Balance/blob/master/DBS%20Balance%20Code%20Final.Rmd. The scoring distributions for the total BESTest, each of its six sections, and for the MDS-UPDRS-III were checked for normality with Shapiro—Wilk tests, both in the ON-medication/ON-stimulation condition and OFF-medication/OFF-stimulation condition. Scores from the ON-medication/ON-stimulation condition and the OFF-medication/OFF-stimulation condition were then compared for the total BESTest to assess our primary aim, for each of its six sections to assess our exploratory aim, and for the MDS-UPDRS-III to characterize the effects of the treatments on motor performance. A paired t-test was used for each of these comparisons if the normality assumption was not violated for either treatment condition. Otherwise, a Wilcoxon signed-rank test was used. For our exploratory aim, a multiple comparisons correction was applied to the resulting probability values for each of the six sections of the BESTest using the Holm–Bonferroni method and the “p.adjust” function from the “stats” package in R [23]. Cohen’s d was calculated for total BESTest score, each of the six sub-sections, and for the MDS-UPDRS-III, to show effect size between the ON-medication/ON-stimulation and OFF-medication/OFF-stimulation conditions.

## 3. Results

Participant characteristics are listed in Table 1. The ON-medication/ON-stimulation condition is represented in Table 1 as “ON,” and the OFF-medication/OFF-stimulation condition is represented as “OFF.” Participants ranged from 12 months to 101 months since STN-DBS implantation.

Mean total BESTest percentage scores and BESTest sub-section percentage scores with standard deviations for the ON-medication/ON-stimulation (ON) and OFF-medication/OFF stimulation (OFF) conditions are presented in Table 2. Probability values from paired t-tests or Wilcoxon signed-rank tests comparing BESTest scores in the ON and OFF conditions are shown in Table 2 in addition to effect sizes.

Total BESTest percentage scores were significantly better in the ON-medication/ON-stimulation condition, compared to the OFF-medication/OFF-stimulation condition. Mean percentage scores for sections (II) Stability Limits/Verticality, (III) Anticipatory Postural Adjustments, (V) Sensory Orientation, and (VI) Stability in Gait were significantly better in the ON-medication/ON-stimulation condition, compared to the OFF-medication/OFF-stimulation condition. Mean percentage scores for sections (I) Biomechanical Constraints and (IV) Postural Responses did not significantly differ between conditions. MDS-UPDRS-III scores were significantly improved in the ON-medication/ON-stimulation condition relative to the OFF-medication/OFF-stimulation condition, as determined by Wilcoxon signed-rank test (*p* < 0.0001, d = 1.46). 

## 4. Discussion

Levodopa and STN-DBS improves overall balance in people with PD, compared to the untreated state (OFF-medication/OFF-stimulation). Levodopa and stimulation, when used in tandem, may affect balance through improved performance in stability limits/verticality, anticipatory postural adjustments, sensory orientation, and stability in gait. Levodopa and stimulation do not appear to change biomechanical constraints or reactive postural responses, at least in the present sample.

The difference in total BESTest scores between conditions represents improvement in overall balance when ON-medication/ON-stimulation was compared to OFF-medication/OFF-stimulation. This indicates that either medication or stimulation, or the combination of the two, improves balance. These findings support our primary hypothesis, and support results from other balance assessments showing that either levodopa or levodopa with STN-DBS improves overall balance [6,7]. However, our study does not distinguish whether medication or STN-DBS alone accounts for the difference in total BESTest scores.

To our knowledge, no existing literature specifically addresses how biomechanical constraints, stability limits/verticality, and sensory orientation respond to levodopa and STN-DBS in people with PD. Our results show that biomechanical constraints do not respond to this combined treatment. This is, perhaps, not surprising as the biomechanical constraints section of the BESTest focuses on standing alignment and lower extremity strength. Additionally, underlying orthopedic issues could limit treatment effects in the biomechanical constraints section. We did find, however, that scores for stability limits/verticality significantly improved when ON-medication/ON-stimulation, compared to OFF-medication/OFF-stimulation, suggesting these treatment strategies improve participants’ abilities to reach and lean to the limits of stability. We also found participants better integrated and responded to sensory feedback from multiple systems when treated with levodopa and STN-DBS, as the scores for the sensory orientation section significantly improved ON-medication/ON-stimulation compared to OFF-medication/OFF-stimulation.

Our results support some previous studies examining the effects of levodopa or STN-DBS on some balance components in people with PD. For example, our results align with a previous report where levodopa improved BBS scores, which largely reflects anticipatory postural adjustments [6]. Our gait stability results from the BESTest also agree with previous reports where levodopa improves gait balance, and that levodopa combined with STN-DBS improves Dual Task Timed Up and Go performance [6,7]. Additionally, a previous report showed that STN-DBS improves automatic postural reaction stability, which is similar to the postural reactions section of the BESTest [11]. However, our results from this section suggest levodopa and STN-DBS do not improve postural responses, as we did not find levodopa and STN-DBS significantly improved scores for this section. This discrepancy could be due to differences in testing procedure, as previous findings were derived from translations delivered by a servo-driven platform, whereas the BESTest relies on manual perturbations by a rater.

It is important to note that even in the optimally treated state (ON-medication/ON-stimulation), balance deficits persist in people with PD with STN-DBS. Leddy et al. proposed a 69% total BESTest cutoff score for identifying fallers in people with PD, with scores below 69% indicating an increased fall risk [16]. Though this cutoff score was not determined specifically for people with STN-DBS, the mean total BESTest score in the current study of 67.6% (SD = 10.9%) in the ON-medication/ON-stimulation condition does fall below this 69% cutoff score. While more work is needed to determine an accurate BESTest cutoff score for identifying fallers specifically in people with STN-DBS, the mean total BESTest score in the current study does appear to reflect balance deficits even in the optimally treated condition. No established normative values for each section of the BESTest exist for this population, but scores from some sections of the BESTest in the current study appear low, even in the optimally treated state. For example, participants had scores lower than 70% in five of the six BESTest sections in the ON-medication/ON-stimulation condition. Though these results require replication on larger samples, these initial results suggest that balance interventions targeting several components of balance may be important for this population.

Several limitations should be noted when interpreting our results. The sample size was relatively small (*n* = 29), participants were highly variable regarding time since DBS surgery, and all participants had stimulation electrodes implanted in the STN. No participants in this study had DBS at other sites, such as the globus pallidus internus (GPi), so no conclusions can be drawn from these results regarding how stimulation at other brain sites may affect balance. Moreover, our aim here was to study people in their most optimal and least optimal states, thus the two treatments were either both OFF or both ON. Therefore, we are not able to determine how medication alone or stimulation alone affects each component of balance. Of note, these findings may be influenced by volunteer bias from two perspectives. Firstly, people with PD and DBS may be reluctant to go OFF-medication/OFF-stimulation, so these results reflect only those willing to withhold treatment for this study. Secondly, participants for this study were recruited to participate in a randomized controlled trial, which would require them to consent to participating in physical therapy twice weekly for eight weeks. As such, the balance performance of those unwilling to participate in a physical therapy program may not be represented in these findings.

Despite the limitations, our results provide novel insight into which specific domains of balance are affected by the current treatment techniques (concurrent STN-DBS and medication) for people with PD. The random order of testing the ON-medication/ON-stimulation and OFF-medication/OFF-stimulation conditions may have helped to avoid the effects of fatigue and learning. Additionally, the rater was blinded to participants’ treatment conditions, minimizing rater bias, although occasionally there was a readily apparent difference in balance performance between conditions. These results represent a crucial first step to more accurately understanding the effects of levodopa and STN-DBS on balance among people with PD. Future work should isolate the effects of each treatment (i.e., medication, stimulation) on individual balance systems, as measured by the BESTest. Further, investigators should longitudinally examine how pre-surgical demographic factors and specific stimulation parameters affect balance over time in people with DBS.

## 5. Conclusions

Levodopa combined with STN-DBS improves overall balance in people with PD. Stability limits/verticality, anticipatory postural adjustments, sensory orientation, and gait stability also significantly improved ON-medication/ON-stimulation compared to OFF-medication/OFF-stimulation. Biomechanical constraints and postural responses did not change significantly between conditions. These results provide the first step to understanding the effects of current treatment strategies on domains of balance in people with PD and STN-DBS.

## Figures and Tables

**Table 1:** Participant Characteristics.

Age in years (mean ± SD)	65.0 ± 6.9
Sex (males, females)	M = 21, F = 8
ON MDS-UPDRS-III score (mean ± SD)	33.0 ± 11.2
OFF MDS-UPDRS-III score (mean ± SD)	49.3 ± 12.4
Years since PD diagnosis (mean ± SD)	11.9 ± 4.7
Months since DBS implantation (mean ± SD)	40.5 ± 30.0
Mini Mental State Examination score (median, [Q1 - Q3])	29.0, [28.0–30.0]
MDS-UPDRS II Score (mean ± SD) ^A^	16.2 ± 7.1
Total levodopa equivalent daily dose (mean ± SD) ^A^	1,020.4 ± 615.4
DBS voltage (left mean/right mean ± left SD/right SD) ^B^	2.7/2.7 ± 0.8/0.8
DBS pulse width (μs) (left mean/right mean ± left SD/right SD) ^B^	61.2/61.2 ± 5.9/5.9
DBS frequency (hz) (left mean/right mean ± left SD/right SD) ^B^	168.1/170.2 ± 25.9/24.9

DBS, deep brain stimulation; MDS-UPDRS, Movement Disorders Society-sponsored revision of Unified Parkinson’s Disease Rating Scale; PD, Parkinson’s disease; Q1, quartile 1; Q3, quartile 3; SD, standard deviation. ^A^ MDS-UPDRS-II and total levodopa equivalent daily dose are derived from n = 28 due to missing data. ^B^ DBS voltage, pulse width, and frequency are derived from n = 26 due to missing data. “Left” refers to left side stimulation and “right” refers to right side stimulation.

**Table 2:** BESTest Scores by Section.

	OFF (mean ± SD)	ON (mean ± SD)	*p*-Value	Cohen’s d
Total BESTest score	59.2 ± 16.5	67.6 ± 10.9	<0.0001 *	0.77
I. Biomechanical Constraints	48.1 ± 24.9	51.7 ± 23.3	0.3543 *	0.22
II. Stability Limits/Verticality	79.2 ± 10.6	86.0 ± 8.6	0.0320 *	0.54
III. Anticipatory Postural Adjustments	57.9 ± 20.1	67.4 ± 10.5	0.0160 *	0.55
IV. Postural Responses	55.0 ± 25.9	63.4 ± 23.4	0.1018	0.38
V. Sensory Orientation	52.2 ± 21.2	60.5 ± 14.8	0.0320 *	0.62
VI. Stability in Gait	57.1 ± 21.1	69.1 ± 16.5	0.0132	0.63

*p*-values for each of the six sub-sections have been adjusted using the Holm–Bonferroni method. * Indicates Wilcoxon signed-rank test. *p*-values are otherwise derived from paired *t*-test.
